# MF-Net: multi-scale feature extraction-integration network for unsupervised deformable registration

**DOI:** 10.3389/fnins.2024.1364409

**Published:** 2024-04-12

**Authors:** Andi Li, Yuhan Ying, Tian Gao, Lei Zhang, Xingang Zhao, Yiwen Zhao, Guoli Song, He Zhang

**Affiliations:** ^1^State Key Laboratory of Robotics, Shenyang Institute of Automation, Chinese Academy of Sciences, Shenyang, China; ^2^Institutes for Robotics and Intelligent Manufacturing, Chinese Academy of Sciences, Shenyang, China; ^3^University of Chinese Academy of Sciences, Beijing, China; ^4^School of Automation and Electrical Engineering, Shenyang Ligong University, Shenyang, China; ^5^Spine Surgery Unit, Shengjing Hospital of China Medical University, Shenyang, China; ^6^Orthopedic Department, The Second Affiliated Hospital of Chongqing Medical University, Chongqing, China

**Keywords:** deformable image registration, unsupervised learning, convolutional neural network, multi-scale, gating mechanism

## Abstract

Deformable registration plays a fundamental and crucial role in scenarios such as surgical navigation and image-assisted analysis. While deformable registration methods based on unsupervised learning have shown remarkable success in predicting displacement fields with high accuracy, many existing registration networks are limited by the lack of multi-scale analysis, restricting comprehensive utilization of global and local features in the images. To address this limitation, we propose a novel registration network called multi-scale feature extraction-integration network (MF-Net). First, we propose a multiscale analysis strategy that enables the model to capture global and local semantic information in the image, thus facilitating accurate texture and detail registration. Additionally, we introduce grouped gated inception block (GI-Block) as the basic unit of the feature extractor, enabling the feature extractor to selectively extract quantitative features from images at various resolutions. Comparative experiments demonstrate the superior accuracy of our approach over existing methods.

## 1 Introduction

Deformable image registration involves obtaining non-rigid spatial transformations from a moving image to a fixed image, representing a crucial step in tasks such as surgical navigation and image-assisted analysis (Nakajima et al., 2020; Drakopoulos et al., 2021; Geng et al., 2024). For instance, Drakopoulos et al. (2021) introduced the deformable registration method into the AR neuro-navigation system to assist brain tumor resection in functional areas of the brain. Geng et al. (2024) used deformable registration to obtain brain templates for Chinese babies, which can be used for investigating neural biomarkers for neurological and neurodevelopmental disorders in Chinese populations. The significance of deformable registration in influencing the outcomes of these tasks cannot be overstated, as it plays a crucial role in ensuring their success.

Learning-based methods for deformable registration involve modeling the registration process as a neural network. This approach entails iteratively optimizing the network parameters across the entire dataset to obtain a shared registration function. Learning-based registration can be categorized into supervised and unsupervised learning methods.

Supervised learning registration uses the true spatial transformations as labels, wherein neural networks are utilized to learn the spatial relationships between moving and fixed images. Obtaining these labels through manual annotation is impractical; hence, they are commonly obtained through traditional algorithms (Cao et al., 2017, 2018; Yang et al., 2017). For instance, Yang et al. (2017) proposed a Large Deformation Diffeomorphic Metric Mapping (LDDMM) model to register brain MR scans by using results from optimizing the LDDMM shooting formulation as labels. Cao et al. (2018) used the SyN algorithm (Avants et al., 2008) and Demons algorithm (Vercauteren et al., 2009; Lorenzi et al., 2013) to obtain displacement fields as labels for training the model, resulting in a model for aligning brain MR scans. However, this method for obtaining labels has limitations. Specifically, the use of traditional algorithms can potentially constrain the model’s performance due to the accuracy limitations inherent in these algorithms. Consequently, the performance of supervised registration is limited by the restrictions of label acquisition.

Due to the limitation of supervised registration, current research has shifted toward unsupervised registration. These models incorporate a differentiable Spatial Transformer Network (STN) module (Jaderberg et al., 2015) to apply the displacement fields generated by neural networks to the moving images, resulting in warped images. The similarity between the warped images and fixed images serves as the loss function guiding the optimization of model parameters (Balakrishnan et al., 2018; Hu et al., 2019; Mok et al., 2020; Ma et al., 2023). VoxelMorph (Balakrishnan et al., 2018), a representative unsupervised registration network, used a U-shaped network as its backbone to align brain MR scans. Huang et al. (2022) proposed a network for brain registration, which enhanced the model’s capabilities by introducing an inception block and a hierarchical prediction block based on the U-shaped network. Additionally, Chen et al. (2022) proposed a brain registration network utilizing transformer modules and adopting a U-shaped structure. The aforementioned work addressed the deformable registration issue to some extent. However, these registration models only extract features from the original resolution image pairs, which overlooks the analysis of multi-scale semantic information and constrains the comprehensive utilization of global and local features by the model. As a result, these methods fail to achieve finer registration.

Several studies have addressed unsupervised registration task from the multi-scale perspective, such as LapIRN (Mok and Chung, 2020), Dual-PRNet (Kang et al., 2022), and Symmetric pyramid network (Zhang et al., 2023). These methods achieve multi-scale registration by progressively warping images through the acquisition of multiple upsampled displacement fields. However, upsampling and composition of displacement fields can lead to error accumulation, resulting in deviation between the final registration outcome and the true transformation, especially when noise or distortions are introduced at multiple stages. In addition, the lack of control over information flow prevents these models from adequately filtering out valid information.

To improve the model’s multi-scale analysis capability, we introduce a new registration network called the multi-scale feature extraction-integration network (MF-Net). This work’s main contributions are:

- Our novel unsupervised deformable registration network is based on a multi-scale feature extraction-integration strategy and comprehensively models both global and detailed information of images, thereby enhancing the deep representation of the registration model. The network is comprised of three main components: an image pyramid, a selective feature extractor (SFE), and a feature integration path (FIP). This design allows for the comprehensive capture of image features at different scales while also integrating them effectively to enhance the overall registration performance.
- The grouped gated inception block (GI-Block) was specifically designed as the basic unit of the SFE in order to facilitate the selective extraction of different features from images of varying resolutions. By employing filters with various receptive fields and utilizing gating mechanism to regulate feature flow, the GI-Block is able to effectively extract quantitative information from images at different resolutions. Furthermore, the implementation of grouped convolution operations within the GI-Block contributes to the efficient processing of information.
- Comparative experiments show that our model achieves higher accuracy than existing models. Ablation studies also confirm the effectiveness of the multi-scale strategy and gating mechanism.

## 2 Methods

### 2.1 Formalized description

For a pair of fixed image F:Ω→R and moving image M:Ω→R defined in the subspace Ω of $R^{3}$ , the objective of deformable registration is to predict a displacement field $\phi :\Omega \to R^{3}$ to warp the moving image so that the warped image M∘ϕ is aligned with the fixed image F , as shown in Equation (1).

(1) $$F\left(x\right)\approx M\left(x+\phi \left(x\right)\right),x\in \Omega$$

Where “ ≈ ” denotes that M∘ϕ and F achieve the highest anatomical similarity, and x denotes any point in the image. We model deformable registration as Equation (2).

(2) $$f_{\theta }\left(F,M\right)=\phi$$

where θ represents the parameters of the function. We employ a neural network to learn this registration function.

### 2.2 Multi-scale feature extraction-integration network (MF-Net)

**Figure 1:**
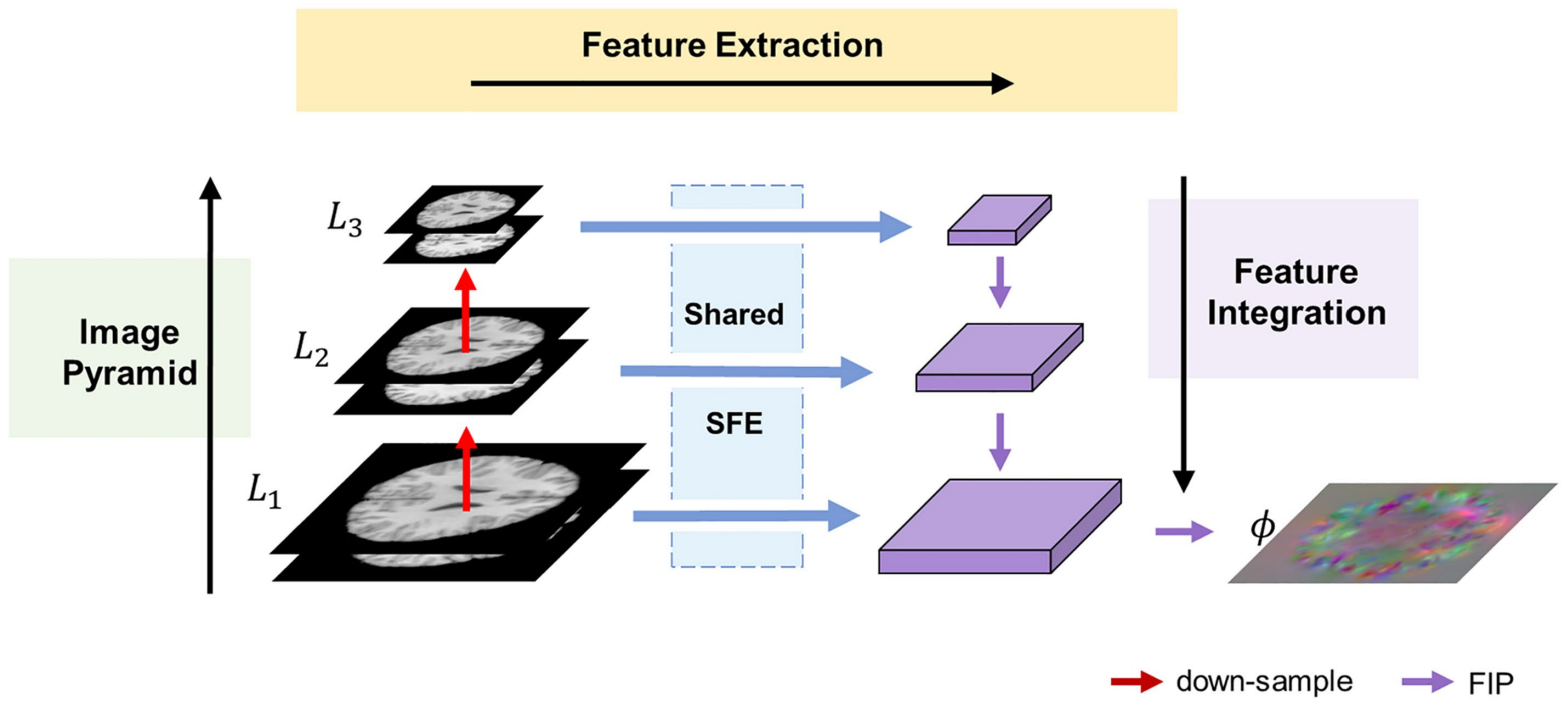
Overview of the proposed MF-Net framework. Our MF-Net consists of three main modules: an image pyramid, a shared SFE, and a FIP. Firstly, the image pyramid is used to create multi-resolution sub-bands of the original image. Then, the shared SFE is employed to extract features from the different sub-bands generated by the pyramid. Finally, the FIP performs the crucial task of integrating the multi-scale features extracted by the SFE and utilizing the integrated features to produce the displacement field.

Figure 1 illustrates the overall architecture of the proposed MF-Net. For clarity, we use 2D slices instead of the original 3D images. Rather than employing an encoder-decoder strategy like U-shaped structure, our method utilizes a multi-scale feature extraction-integration strategy. Specifically, our model is composed of an image pyramid, a selective feature extractor (SFE), and a feature integration path (FIP). To begin, an image pyramid is generated from an image pair consisting of a fixed image F and a moving image M . Following this, the different levels of the image pyramid are input into a shared SFE to extract features at corresponding scales. Ultimately, the extracted multi-scale features are integrated by FIP to generate the displacement field ϕ , which includes the displacement of each pixel in the *x*, *y*, and *z* directions.

#### 2.2.1 Image pyramid

To address the limitations of the U-shaped structure, which only extracts features from the original resolution images, an image pyramid component is introduced into our network. This component follows the multi-resolution strategy employed in traditional image algorithms. Specifically, the fixed image and the moving image are concatenated along the channel dimension and down-sampled using trilinear interpolation to generate an N-layer image pyramid $\left\{L_{1},L_{2},\ldots ,L_{N}\right\}$ , where $L_{1}$ is the original image pair. For simplicity, N is set to 3 in this paper.

#### 2.2.2 Selective feature extractor

To adaptively extract quantitative information from various levels of the image pyramid, we propose the SFE. The SFE utilizes grouped gated inception blocks (GI-Blocks) with a gating mechanism, allowing for adaptive feature extraction from images at varying resolutions. For various levels of the image pyramid, features are extracted using a shared SFE. This design ensures versatile feature extraction capabilities tailored to the varying resolutions of the image pyramid.

##### 2.2.2.1 Architecture of SFE

**Figure 2:**
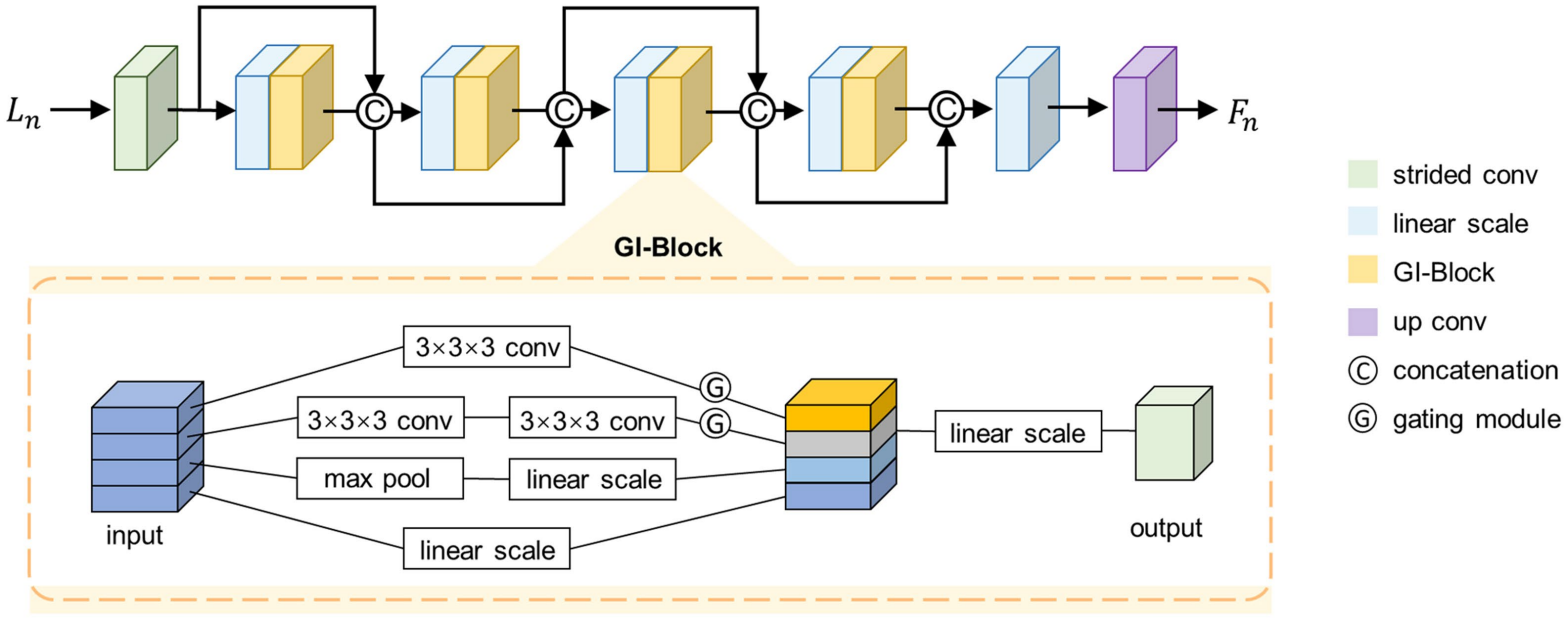
Architecture of the SFE. For simplicity, only four GI-Blocks are shown.

The proposed SFE architecture is shown in the top half of Figure 2. SFE is comprised of densely connected GI-Blocks. The decision to use dense connections for feature extraction is rooted in the idea that these connections continually amalgamate features at various levels, thereby allowing the model to seamlessly integrate semantic information from different levels and synthesize semantic cues for the generation of a registration displacement field.

We start by feeding a specific level $L_{n}$ from the set $\left\{L_{1},L_{2},\ldots ,L_{N}\right\}$ into a strided convolutional layer to halve the size of the feature map, as shown in Equation (3).

(3) $$Y_{n}^{1}=\mathrm{StridedCon}v_{3\times 3\times 3}^{2\to C_{1}}\left(L_{n}\right)$$

Where $\mathrm{StridedCon}v_{3\times 3\times 3}^{2\to C_{1}}$ represents a 3 × 3 × 3 kernel size convolutional layer with input channels of two, output channels of $C_{1}$ , and a stride of two. Next, the feature map is fed into a dense path comprised of densely connected GI-Blocks, as shown in Equation (4).

(4) $$Y_{n}^{2}=\mathrm{DensePath}\left(Y_{n}^{1}\right)$$

Where the DensePath represents a densely connected path consisting of M GI-Blocks. We fix the output channel number of the GI-Blocks as K , which is also referred to as the growth rate (Huang et al., 2017).

According to the structure of the dense connection, the channel number of $Y_{n}^{2}$ is $C_{1}+M\times K$ . To simultaneously fix the input channel number of the GI-Blocks, we linearly scale the channel number of the feature map to 4K before feeding it into the GI-Block. Finally, we linearly scale the channel number of the output from the densely connected path to 4K and feed it into a transposed convolutional layer with an output channel number of $C_{2}$ to restore the size of the feature map, as shown in Equations (5, 6).

(5) $$Y_{n}^{3}=\mathrm{LinearScal}e^{\left(C_{1}+M\times K\right)\to 4K}\left(Y_{n}^{2}\right)$$

(6) $$F_{n}=\mathrm{TransposeCon}v_{4\times 4\times 4}^{4K\to C_{2}}\left(Y_{n}^{3}\right)$$

We set K and $C_{1}$ to 32, $C_{2}$ to 16, and M to 5. Note that for simplicity, only four GI-Blocks are shown in Figure 2.

##### 2.2.2.2 GI-Block

To adaptively extract quantitative information from images at various resolutions, we propose the GI-Block. The structure of the GI-Block is shown in the lower part of Figure 2. The proposed GI-Block consists of four parallel branches. The first branch employs a 3 × 3 × 3 convolutional layer to extract features with a smaller receptive field. The second branch uses two 3 × 3 × 3 convolutional layers to approximate a 5 × 5 × 5 convolution (Szegedy et al., 2016), extracting features with a larger receptive field. The third branch includes a max-pooling layer and a linear scaling layer (i.e., a 1 × 1 × 1 convolutional layer). The max-pooling layer is responsible for extracting representative information from the input feature map, and the linear scaling layer scales the extracted representative information. Finally, the fourth branch utilizes only a linear scaling layer to preserve the features of the original input. We split the input feature map into four parts along the channel dimension, and then input each part into each of the four branches mentioned above.

To enhance the differentiation of receptive field weights for feature maps at varying resolutions in GI-Block, we introduce the gating mechanism. This mechanism addresses the need for distinct receptive field weights for images with different resolutions. Specifically, information extracted from a smaller image should include more features extracted using a smaller receptive field filter, while information extracted from a larger image should include more features extracted using a larger receptive field filter. To achieve this, the gating mechanism is incorporated. We feed the features extracted by the first two branches into a convolutional layer with a kernel size of 3 × 3 × 3 and an activation function of SoftSign to obtain weights in the range of 0–1. These weights are then multiplied with the original features, resulting in the gated features. The formula for the gating mechanism is described as Equation (7).

(7) $$Y=X\times \mathrm{Sigmoid}\left(\mathrm{Con}v_{1\times 1\times 1}^{C\to C}\left(X\right)\right)$$

Where *X* represents the input to the gating mechanism, and *Y* represents the output of the gating mechanism.

Finally, the feature maps extracted by different branches are merged along the channel dimension and fused through a 1 × 1 × 1 convolutional layer to prevent potential feature disintegration caused by group convolution.

#### 2.2.3 Feature integration path

**Figure 3:**
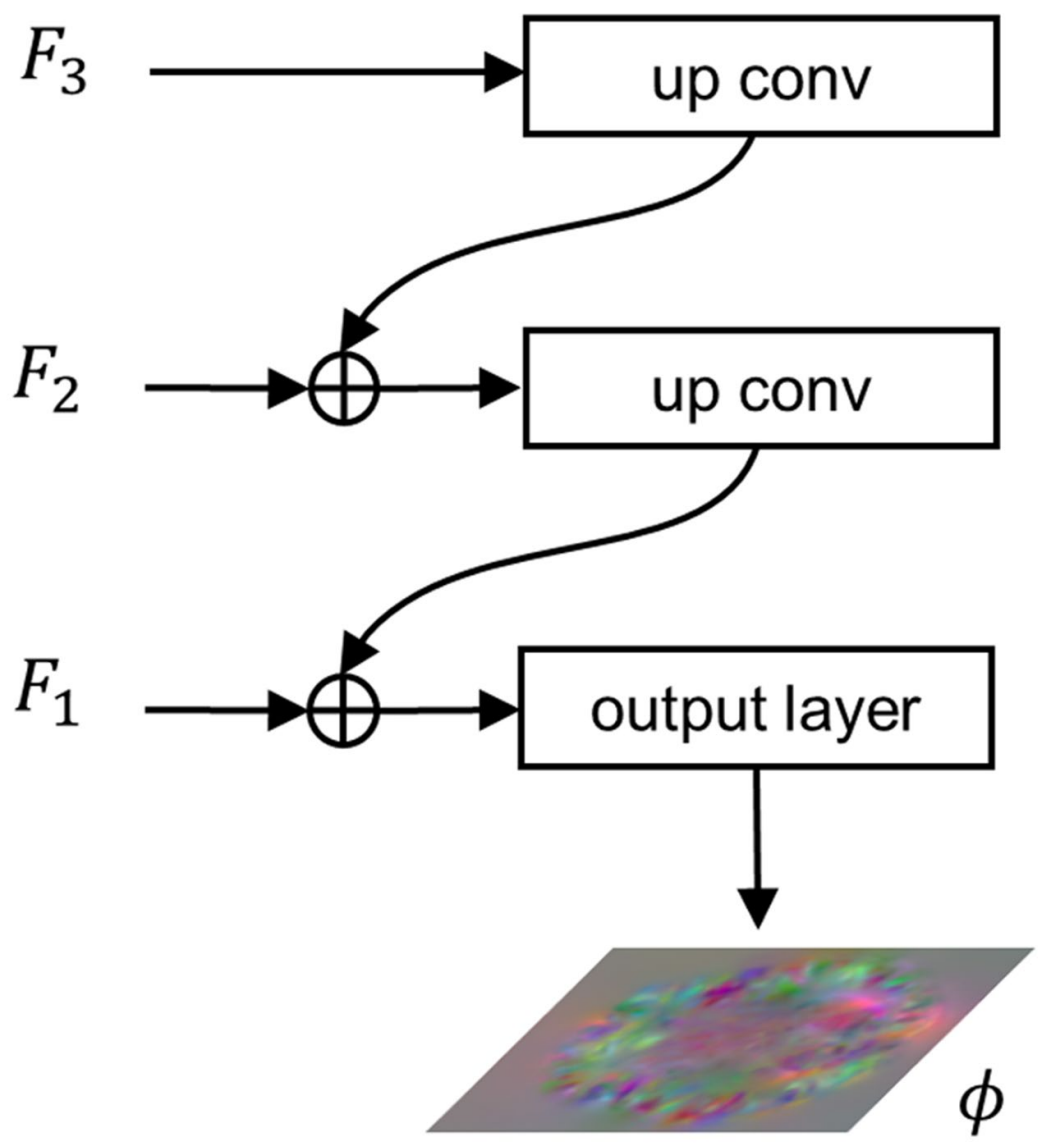
Architecture of the FIP. $F_{1}$ , $F_{2}$ , and $F_{3}$ represent features extracted from $L_{1}$ , $L_{2}$ , and $L_{3}$ , respectively. ϕ denotes the final output of the network, i.e., the displacement field.

To integrate the extracted multi-scale semantic information and generate a registration displacement field using the integrated semantic information, we propose the FIP module. Figure 3 illustrates the structure of the FIP. The lower resolution feature map is doubled in size through transpose convolution and then connected to the feature map at a higher resolution via residual connection. The resulting feature map then undergoes the same process iteratively until reaching the feature map at the highest resolution, as shown in Equation (8).

(8) $$F_{n}^{\prime }=\mathrm{TransposeCon}v_{4\times 4\times 4}^{C2\to C2}\left({F_{n+1}}^{\prime }\right)+F_{n},n\in \left[1,\;N-1\right]$$

When n=N , $F_{N}^{\prime }=F_{N}$ . Finally, the integration features pass through the output layer, a convolutional layer with a SoftSign activation function, to produce the registration flow field, as shown in Equation (9).

(9) $$\phi =R\times \mathrm{SoftSign}\left(\mathrm{Con}v_{3\times 3\times 3}^{C\to 3}\left(F_{N}^{\prime }\right)\right)$$

where R is the scale factor and we set R to 20.

### 2.3 Loss functions

To guide the optimization of the neural network, we employ an intensity-based similarity metric between M∘ϕ and F . Our method is unsupervised as the loss function does not necessitate the introduction of labels. In order to mitigate folding in the displacement field that deviates from anatomical constraints, we utilize the gradient norm of the displacement field as a regularization term.

#### 2.3.1 Similarity loss

We use normalized cross-correlation (NCC) to measure the similarity between M∘ϕ and F . The NCC function yields values ranging from 0 to 1, with higher values indicating higher similarity. We take the negative of the similarity metric so that as the loss function decreases, the similarity measure between the images increases, as shown in Equation (10).

(10) $$L_{sim}\left(M\circ \phi ,F\right)=-NCC\left(M\circ \phi ,F\right)$$

#### 2.3.2 Grad loss

If the optimization of the neural network is guided solely by the similarity metric between M∘ϕ and F , it may lead to results that do not conform to anatomical constraints, such as abrupt changes or folding of the displacement field. To mitigate this situation, we introduce the norm of the displacement field gradient as a regularization term in the loss function, as shown in Equation (11).

(11) $$L_{\mathrm{grad}}\left(\phi \right)=\frac{1}{\left|3\Omega \right|}\underset{x\in \Omega }{\sum }{\left\|\nabla \phi \left(x\right)\right\|}^{2}$$

We combine the similarity metric and the regularization term into the overall loss function, as shown in Equation (12).

(12) $$L_{\mathrm{total}}=L_{sim}\left(M\circ \phi ,F\right)+\lambda L_{\mathrm{grad}}\left(\phi \right)$$

Where λ is a hyperparameter used to balance the contributions of the two terms.

## 3 Experiments

### 3.1 Dataset and preprocessing

We conducted atlas-based registration experiments on the publicly available OASIS dataset (Marcus et al., 2007). OASIS comprises 416 3D brain MR scans from participants aged 18–96. We utilized a processed version of OASIS (Balakrishnan et al., 2019), where the brain scans underwent skull stripping and subcortical structure segmentation. For our experiments, we randomly selected 200, 35, and 35 scans as the training, validation, and test sets, respectively. We randomly chose five scans from each of the validation set and test set as fixed images, with the remaining scans serving as moving images. That is, each method was optimized on a training set containing 10 × 200 image pairs during training, and each method registered 5 × 30 image pairs during validation or testing.

We cropped unnecessary regions around the brain and resample the images to 96 × 112 × 96. Subsequently, intensity normalization was applied to each scan, mapping pixel intensities to the range [0,1] to facilitate network convergence. Finally, we conducted affine pre-registration on the moving and fixed images in the dataset using ANTs toolkit (Avants et al., 2011).

### 3.2 Baseline methods and implementation

We compared the proposed MF-Net with three baseline methods, namely VoxelMorph, SYMNet (Mok et al., 2020), and LapIRN. VoxelMorph is a classic unsupervised registration model utilizing a U-shaped convolutional network to predict the displacement field. We evaluated two variants proposed in their paper: VoxelMorph-1 and VoxelMorph-2. SYMNet predicts both forward and inverse transformations simultaneously through a U-shaped network, and provides diffeomorphic properties. LapIRN combines displacement fields at multiple scales to obtain the final registration displacement field. This study also predicts diffeomorphic transformations. We conducted evaluation on both LapIRN and its variant, LapIRN_disp.,_ the latter of which abandons the diffeomorphic property while enhancing registration accuracy. All the mentioned methods were used for brain MR registration in their respective original papers. We used the official implementations of these methods and followed the recommended guidelines, adjusting hyperparameters to ensure the best registration performance.

We implemented MF-Net using PyTorch (Paszke et al., 2017) and employed the AdamW optimizer (Loshchilov and Hutter, 2017) with a learning rate of 0.0001 for training over 100 epochs. The hyperparameter λ is set to 1. All experiments were conducted on a personal workstation equipped with an RTX 3080 GPU and an Intel(R) i7-10700KF CPU.

### 3.3 Evaluation metrics

#### 3.3.1 Dice score

We quantified the degree of overlap between the fixed image and the warped image using the dice score, Dice (1945) computed from the anatomical tissue segmentation masks of the fixed image and the warped image, as shown in Equation (13).

(13) $$\mathrm{Dice}=2\cdot \frac{\left|F^{msk}\cap \left(M^{msk}\circ \phi \right)\right|}{\left|F^{msk}\right|+\left|M^{msk}\circ \phi \right|}$$

Where $F^{msk}$ and $M^{msk}$ denote the subcortical segmentation masks of the fixed image and the moving images, respectively. The dice score, ranging from 0 to 1, signifies the degree of overlap, with a higher score reflecting increased registration accuracy.

#### 3.3.2 Jacobian determinant

We evaluated the smoothness of the deformation field by computing the percentage of voxels with a non-positive Jacobian determinant (|JD ≤ 0|). The formula for the Jacobian determinant of the displacement field is given by Equation (14).

(14) $$J_{\phi }\left(p\right)=|\begin{array}{ccc} \frac{\partial \phi_{x}\left(p\right)}{\partial x} & \frac{\partial \phi_{x}\left(p\right)}{\partial y} & \frac{\partial \phi_{x}\left(p\right)}{\partial z}\\ \frac{\partial \phi_{y}\left(p\right)}{\partial x} & \frac{\partial \phi_{y}\left(p\right)}{\partial y} & \frac{\partial \phi_{y}\left(p\right)}{\partial z}\\ \frac{\partial \phi_{z}\left(p\right)}{\partial x} & \frac{\partial \phi_{z}\left(p\right)}{\partial y} & \frac{\partial \phi_{z}\left(p\right)}{\partial z} \end{array}|$$

A smaller percentage suggests a higher level of smoothness.

### 3.4 Comparative evaluation

Table 1 provides the average dice score and the percentage of voxels with non-positive Jacobian determinants (|JD ≤ 0|) for all subjects and structures, encompassing VoxelMorph-1, VoxelMorph-2, SYMNet, LapIRN, LapIRN_disp_, and our MF-Net. We also include affine transformation for comparison purposes. It is evident that our MF-Net achieves better registration accuracy with few folding voxels. While SYMNet and LapIRN achieved entirely smooth displacement fields through diffeomorphic transformation, this achievement comes at the expense of registration accuracy.

**Table 1:** Comparison of different methods on the dataset, with affine registration used for reference.

Method	Dice (%)	|JD ≤ 0|
Affine Only	56.33 ± 0.04	-
VoxelMorph-1	73.07 ± 0.04	186 ± 38
VoxelMorph-2	73.94 ± 0.05	392 ± 69
SYMNet	71.89 ± 0.31	0.5 ± 0.4
LapIRN	71.43 ± 0.04	**0**
LapIRN_disp_	74.89 ± 0.18	1757 ± 259
MF-Net (ours)	**75.38** ± **0.05**	332 ± 22

Dice measures registration accuracy (higher values are better), and |JD ≤ 0| indicates the number of folding voxels that do not conform to the anatomical structure. Please note that the best-performing results are highlighted in bold.

**Figure 4:**
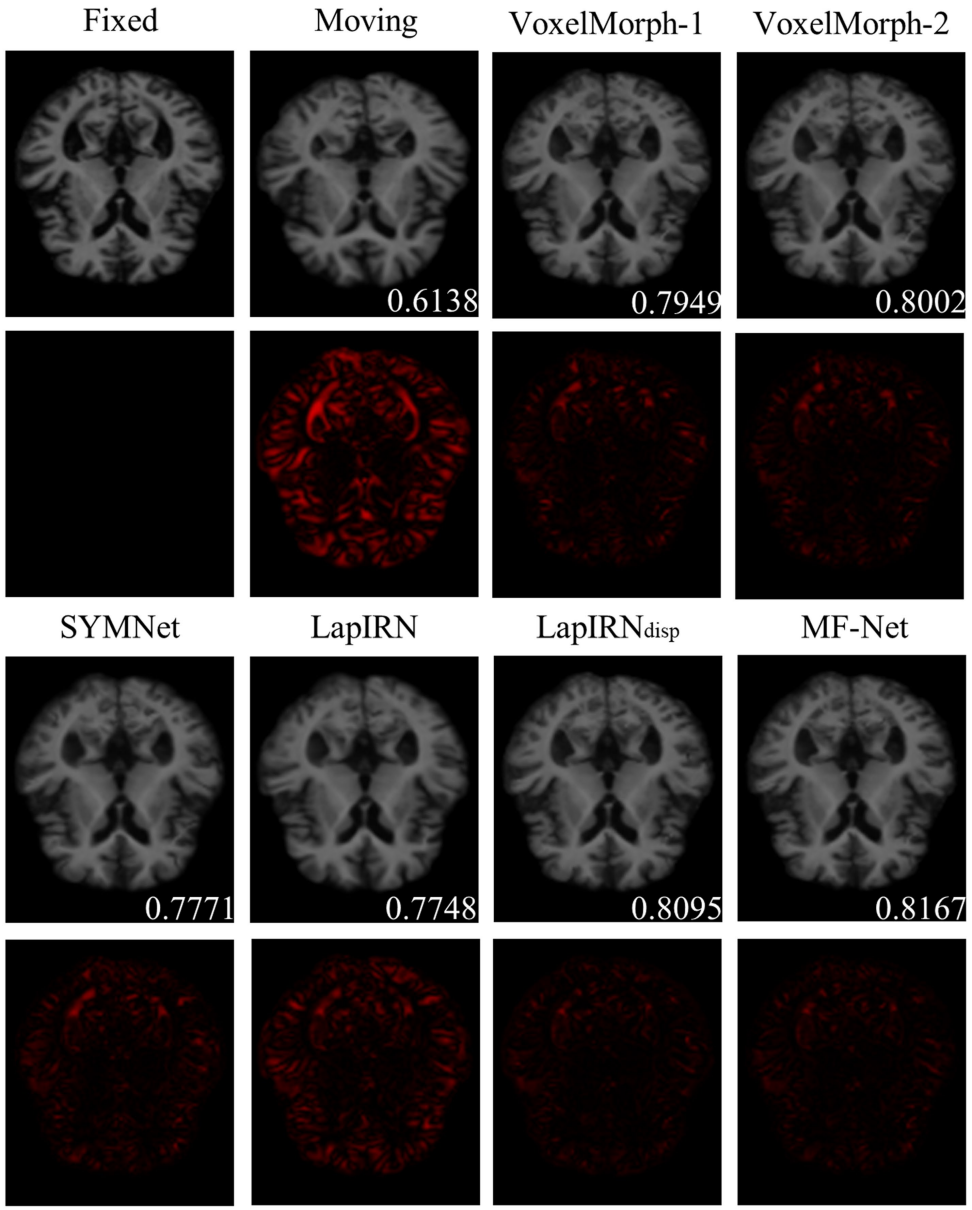
The registration results for a representative sample within the dataset employing six distinct methods. The second and fourth rows show the heat maps, which illustrate the absolute differences between the warped image and the fixed image. Notably, the lower right corner of the warped image shows the dice score, which indicates the degree of similarity between the warped image and the fixed image.

**Figure 5:**
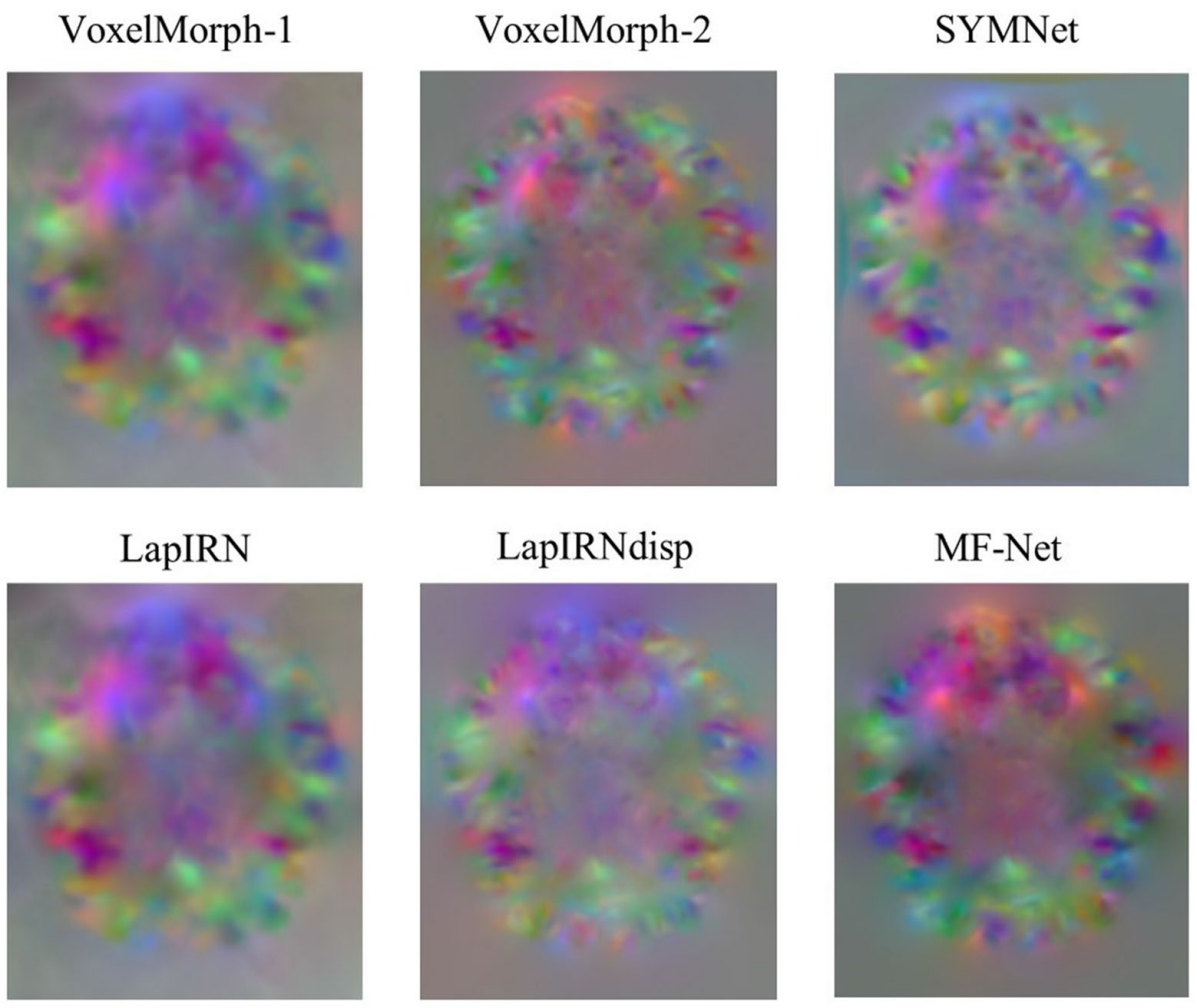
Slices of the displacement field. The red, green, and blue colors in the image show voxel displacement in three directions.

Figure 4 illustrates registration example slices of brain MR scans under different methods. As evident from the difference map between the fixed image and the warped image obtained by various methods, our method yielded a warped image that is most similar to the fixed image. Both quantitative and qualitative evaluations demonstrate the effectiveness of our multi-scale feature extraction-fusion strategy. Additionally, to improve comprehension of the registration process, we display the slices of the displacement field output by each method in Figure 5.

### 3.5 Ablation analysis

To further validate the effectiveness of the multi-scale feature extraction-fusion strategy, we omitted the multi-scale strategy of MF-Net and predicted the displacement field solely based on images at the original resolution. We label this network as MF-Net-1. Table 2 displays the registration metrics of MF-Net and MF-Net-1 on the test set. It can be observed that MF-Net exhibits higher registration accuracy than MF-Net-1. This experiment demonstrates that our network, employing the multiscale analysis strategy, can more efficiently capture features at various scales, thereby improving the model’s registration performance.

**Table 2:** Ablation analysis of the multiscale strategy on the MF-Net.

Method	Dice (%)	|JD ≤ 0|
MF-Net	75.38 ± 0.05	332 ± 22
MF-Net-1	75.18 ± 0.01	268 ± 11

MF-Net is the proposed model, and MF-Net-1 is based on MF-Net but eliminates the multi-scale feature extraction-integration strategy.

To verify the effectiveness of the proposed gating mechanism, we omitted the gating mechanism of GI-Block in our variant MF-Net-2. Table 3 presents the quantitative evaluation results before and after the removal. It is evident that MF-Net demonstrates better registration accuracy compared to MF-Net-2. This experiment demonstrates that the gating mechanism can efficiently extract meaningful information from redundant cascade features, automatically learning the weights of different sensory field features, and thereby improving the model’s registration performance.

**Table 3:** Ablation analysis of the gating mechanism on the MF-Net.

Method	Dice (%)	|JD ≤ 0|
MF-Net	75.38 ± 0.05	332 ± 22
MF-Net-2	75.02 ± 0.05	168 ± 8

MF-Net is the proposed model, and MF-Net-2 is based on MF-Net but eliminates the gating mechanism.

## 4 Discussion

Although both utilize multi-scale information from images, MF-Net differs from existing models represented by LapIRN. Like most existing registration networks based on multi-scale strategies, LapIRN achieves multi-scale information fusion by continuously compositing the generated multi-scale displacement fields. In contrast, MF-Net extracts multi-scale features, then fuses these features, and finally, obtains the registration displacement field from the fused features. In other words, MF-Net fuses the multi-scale information earlier than LapIRN, which may be one of the reasons for the better accuracy of our method, considering that LapIRN uses multiple displacement fields that may cause the accumulation of errors. Furthermore, our feature extractor adjusts the flow of feature information through gating mechanism, which may be another contributing factor.

In addition, we changed the resolution of images in the preprocessing stage through resampling, potentially impacting the model’s performance due to the loss of image information. It is important to note that while our manipulation has affected the results of individual models, it does not alter the comparison of different models, as our comparisons of different models were conducted under the same conditions. The disparity between MF-Net and the baselines might become more apparent when training and testing are conducted using images at their original resolution. Given our model’s better feature extraction abilities, it is expected to more effectively analyze the additional information available at the original resolution. Therefore, the gap between our model and the baselines may expand in such scenarios.

## 5 Conclusion

In this study, we introduced a novel 3D image deformation registration network named MF-Net, which is built upon the multi-scale feature extraction-fusion strategy. MF-Net enhances the model’s analytical ability by integrating multi-scale information, thereby balancing image texture and detail registration. Within our network, we design the GI-Block as the basic unit of the feature extractor, which adaptively extracts quantitative information through gating mechanism. Compared with existing registration approaches, our network demonstrated better registration accuracy. Ablation experiments further indicated that the proposed multi-scale strategy can improve registration performance. Our work has potential applications in the fields of neuronavigation and brain image-assisted analysis. This expands the scope for future research and applications in the realms of neurosurgery and neuroscience.

## Data availability statement

Publicly available datasets were analyzed in this study. This data can be found here: https://www.oasis-brains.org/.

## Author contributions

AL: Writing – review & editing, Writing – original draft, Software, Methodology, Conceptualization. YY: Visualization, Writing – review & editing, Writing – original draft. TG: Writing – review & editing, Writing – original draft. LZ: Writing – review & editing, Writing – original draft. XZ: Writing – review & editing, Writing – original draft. YZ: Writing – review & editing, Writing – original draft. GS: Supervision, Resources, Funding acquisition, Writing – review & editing, Writing – original draft. HZ: Investigation, Visualization, Writing – review & editing.
